# Cytokine Plasma Levels: Reliable Predictors for Radiation Pneumonitis?

**DOI:** 10.1371/journal.pone.0002898

**Published:** 2008-08-06

**Authors:** Claudia E. Rübe, Jan Palm, Michael Erren, Jochen Fleckenstein, Jochem König, Klaus Remberger, Christian Rübe

**Affiliations:** 1 Department of Radiotherapy and Radiooncology, Saarland University, Homburg/Saar, Germany; 2 Institute of Clinical Chemistry and Laboratory Medicine, University of Münster, Münster, Germany; 3 Institute of Medical Biostatistics, Epidemiology and Informatics, Johannes Gutenberg University, Mainz, Germany; 4 Department of Pathology, Saarland University, Homburg/Saar, Germany; Dresden University of Technology, Germany

## Abstract

**Background:**

Radiotherapy (RT) is the primary treatment modality for inoperable, locally advanced non-small-cell lung cancer (NSCLC), but even with highly conformal treatment planning, radiation pneumonitis (RP) remains the most serious, dose-limiting complication. Previous clinical reports proposed that cytokine plasma levels measured during RT allow to estimate the individual risk of patients to develop RP. The identification of such cytokine risk profiles would facilitate tailoring radiotherapy to maximize treatment efficacy and to minimize radiation toxicity. However, cytokines are produced not only in normal lung tissue after irradiation, but are also over-expressed in tumour cells of NSCLC specimens. This tumour-derived cytokine production may influence circulating plasma levels in NSCLC patients. The aim of the present study was to investigate the prognostic value of TNF-α, IL-1β, IL-6 and TGF-β1 plasma levels to predict radiation pneumonitis and to evaluate the impact of tumour-derived cytokine production on circulating plasma levels in patients irradiated for NSCLC.

**Methodology/Principal Findings:**

In 52 NSCLC patients (stage I–III) cytokine plasma levels were investigated by ELISA before and weekly during RT, during follow-up (1/3/6/9 months after RT), and at the onset of RP. Tumour biopsies were immunohistochemically stained for IL-6 and TGF-β1, and immunoreactivity was quantified (grade 1–4). RP was evaluated according to LENT-SOMA scale. Tumour response was assessed according to RECIST criteria by chest-CT during follow-up. In our clinical study 21 out of 52 patients developed RP (grade I/II/III/IV: 11/3/6/1 patients). Unexpectedly, cytokine plasma levels measured before and during RT did not correlate with RP incidence. In most patients IL-6 and TGF-β1 plasma levels were already elevated before RT and correlated significantly with the IL-6 and TGF-β1 production in corresponding tumour biopsies. Moreover, IL-6 and TGF-β1 plasma levels measured during follow-up were significantly associated with the individual tumour responses of these patients.

**Conclusions/Significance:**

The results of this study did not confirm that cytokine plasma levels, neither their absolute nor any relative values, may identify patients at risk for RP. In contrast, the clear correlations of IL-6 and TGF-β1 plasma levels with the cytokine production in corresponding tumour biopsies and with the individual tumour responses suggest that the tumour is the major source of circulating cytokines in patients receiving RT for advanced NSCLC.

## Introduction

Radiotherapy (RT) is the most important treatment modality for inoperable, locally advanced non-small-cell lung cancer (NSCLC), but conventional RT with doses in the range of 60–70 Gy is often insufficient to eradicate the tumour, with local control rates of only 10–20% at one year [1], [2]. RT dose escalation is a promising approach, but is limited by the low tolerance of the surrounding normal lung tissue. Radiation pneumonitis (RP), which represents the acute phase of radiation-induced lung injury, is the major dose-limiting toxicity in radiotherapy for NSCLC. The symptoms of RP range from dyspnea, cough, and fever to death from respiratory failure. About 10–15% of NSCLC patients develop severe lung toxicity after thoracic irradiation, and a notable percentage of these patients die from RP [3], [4], [5].

Although the pathogenesis of RP remains unclear, this complex inflammatory process involves an interplay of cellular interactions between lung parenchymal cells and circulating immune cells mediated through a variety of cytokines, chemokines, adhesion molecules, etc.[6], [7], [8]. Animal data have shown an early overproduction of both pro-inflammatory and pro-fibrogenic cytokines during thoracic irradiation and have suggested a role of the sustained production of these cytokines in the development of acute and late pulmonary toxicities [9], [10], [11], [12], [13], [14]. In humans, some clinical reports have shown changes in the plasma concentrations of TGF-β1 and IL-6 during RT and suggested that these variations could identify patients at risk of RP [15], [16], [17], [18], [19], [20], [21], [22].

However, cytokines are also produced in tumours, by the tumour cells themselves or by host stromal and immune cells of the tumour microenvironment [23], [24] This tumour-derived cytokine production may influence the circulating plasma levels in cancer patients and may therefore confuse the results when investigating plasma markers for RP. There is increasing evidence that a wide variety of cytokines are involved at every stage of the tumourigenic process [23], [24]. Especially, the well-established TGF-β pathways regulate an important network of signals that have pivotal effects on the carcinogenic process. TGF-β plays a central role in the control of cellular growth, differentiation and apoptosis and many tumors escape the inhibitory function of TGF-β through mutational inactivation of genes within the TGF-β signalling network [25], [26]. During tumour progression, TGF-β induces tumour invasion and metastasis through increasing cell motility [27]. Tumour cell migration is a complex process which relies on partial degradation of basement membranes as well as specific integrin signalling, which enable cancer cells to detach from neighbouring cells [28]. TGF-β and other cytokines regulate the production and activity of proteases, capable of breaking down components of the extracellular matrix, and therefore promoting the migratory activity of tumour cells [9], [29]. Moreover, TGF-β promotes tumor vascularisation by recruiting angiogenic factors such as vascular endothelial growth factor (VEGF) known to activate a signalling cascade triggering angiogenesis [30], [31]. Another tumour promoting effect is the TGF-β mediated suppression of immunosurveillance. TGF-β has been shown to negatively regulate the functions of both cytotoxic T-cells and natural killer cells allowing tumours to evade immunologic cell death [32]. The pro-inflammatory cytokines TNF-α, IL-1 and IL-6, by contrast, may promote cancer progression by a variety of other mechanisms, that are only now beginning to be elucidated [23], [33]. Moreover, TNF-α is involved in the complex and poorly understood process of cancer-induced cachexia or muscle wasting [34]. In summary, cytokines play a critical role in promoting tumour cell growth, attenuating apoptosis, and facilitating angiogenesis, invasion and metastasis. The increased production of cytokines in tumour tissues which is launched into the blood circulation suggests the possibility of using cytokine plasma levels as tumour markers to monitor the disease course. Actually, several clinical studies proposed the use of cytokine plasma measurements to monitor the therapeutic effect of RT in patients with NSCLC [35], [36].

Against this background of controversial data, the present study was designed to investigate the prognostic value of TNF-α, IL-1β, IL-6 and TGF-β1 plasma levels to predict RP and to evaluate the potential impact of a tumour-derived cytokine production on circulating plasma levels in patients irradiated for NSCLC. For this purpose, the cytokine plasma levels assessed before and during RT were correlated with the incidence of RP. Moreover, for each individual patient, the cytokine plasma levels measured before RT were correlated with the cytokine production in the corresponding tumour biopsies and cytokine plasma levels measured after RT were correlated with the individual tumour responses of the patients during follow-up.

## Materials and Methods

### Patient eligibility

Patients with medically inoperable or locally advanced NSCLC who received radiotherapy (RT) or radio-/chemotherapy (RCT) in our department between January 2002 and January 2005 were enrolled onto this prospective study. Inclusion criteria were as follows: histologically confirmed NSCLC; medically inoperable or locally advanced NSCLC (stage I–III); no previous radiotherapy given; Karnofsky performance status ≥70%; life expectancy ≥6 months. Protocol procedures were approved by the local ethics committee, and all patients provided written informed consent.

### Treatment description

Computed tomography (CT) based three-dimensional dose computations were performed for all patients using the treatment planning system ADAC Pinnacle. Target volumes (i.e. gross tumor volume [GTV], planning target volume [PTV]) were defined according to ICRU-50 report. PTV encompassed all visible local and regional disease with a 1–2 cm margin based on the thoracic CT-scan performed prior to the start of treatment. Lungs were automatically contoured, excluding the GTV. Dose volume histograms (DVH) of the lungs (considered as combined-paired organ) were calculated based on CT-derived lung volumes. The mean lung dose (MLD), as well as V20 and V30 values (irradiated lung volume receiving more than 20 and 30 Gy, respectively) were calculated from lung DVHs.

Thoracic RT was delivered using a linear accelerator with 6MV and/or 23MV photons. 28 patients received curative cisplatin-based radio-/chemotherapy (RCT): 14 patients received concurrent cisplatin (80 mg/m^2^/d), vinorelbine (15 mg/m^2^/d) and normo-fractionated radiotherapy of 66 Gy according to the phase II study of Vokes *et al.* [37]. 14 patients received concurrent cisplatin (50 mg/m^2^/d), etoposide (50 mg/m^2^/d) and normo-fractionated radiotherapy of 60 Gy according to the phase II study of Albain *et al.* [38]. 14 patients not eligible for platinum-based chemotherapy were treated with normo-fractionated radiotherapy alone (66 Gy) (RT). 10 patients were treated according to a palliative accelerated irradiation regimen (PAIR) with a total dose of 32 Gy, applied in two daily fractions of 2 Gy with a time interval of 6 hours, in a total treatment time of 10 days [39].

### Circulating cytokine analysis

In each patient, serial plasma samples for TNF-α, IL-1β, IL-6 and TGF-β1 analysis were obtained before RT, once (RT/RCT) or twice (PAIR) weekly during RT, and at every follow-up examination (1, 3, 6 and 9 months after RT). In addition, cytokine plasma levels were measured at the onset of symptomatic RP. To avoid platelet degranulation, all blood samples were taken without a tourniquet, collected in EDTA tubes, immediately chilled on ice, and then centrifuged within 15 min of collection. TNF-α, IL-1β, IL-6 and TGF-β1 concentrations were measured by specific enzyme-linked immunoassay (ELISA) kits (Quantikine™, R&D Systems, Minneapolis, MN, USA) according to manufacturer's protocol. As latent TGF-β1 was activated to the immunoreactive form by acidification, total TGF-β1 (active and latent form) was measured. Cytokine values determined for normal controls (plasma samples of 20 healthy individuals) were in concordance with previously published data from other laboratories investigating more than 400 healthy individuals with the same ELISA kits [40]. The mean control values +2SD were regarded as cut-off between normal and pathologically elevated cytokine levels (cut-off values: TNF-α: 2.8 pg/ml; IL-1β: 1.63 pg/ml, IL-6: 12.5 pg/ml; TGF-β1: 150 pg/ml). In total, 480 plasma samples were analysed for TNF-α, IL-1β, IL-6 and TGF-β1. All measurements were done in duplicate and mean values were used in further analysis.

### Immunohistochemical staining for TGF-β1 and IL-6 in tumour specimens

Tumour specimens (obtained by needle biopsies before treatment) were fixed in formalin, paraffin-embedded, and sectioned at an average thickness of 5 µm. After dewaxing in xylene and rehydration in graded alcohols, sections were pre-incubated with H_2_O_2_ and blocked with rabbit serum (Chemical Credential, Aurora, Ohio, USA). Afterwards, sections were incubated with chicken-anti-human TGF-β1 antibody or goat-anti-human IL-6 antibody (R&D Systems; 1∶30 dilution) followed by biotinylated secondary antibody (rabbit-anti-chicken, Biomol, Hamburg, Germany, 1∶1000; rabbit-anti-goat, Dako, Glostrup, Denmark; 1∶200). Sections were labelled by avidin-biotin-peroxidase complex (Dako), followed by diaminobenzidine (DAB, Sigma-Aldrich, Taufkirchen, Germany) development. Finally, sections were counterstained with haematoxylin and mounted in Entellan (Merck, Darmstadt, Germany).

The immunoreactivity for IL-6 and TGF-β1 in the lung cancer specimens was quantified using previously validated procedures [41], [42]: no staining (grade 0), weak but detectable focal staining (grade 1), moderate staining (grade 2), marked staining (grade 3), very intense staining of the tumour cells (grade 4). All lung cancer specimens were subjected to anonymous evaluation, whereby 5–10 non-overlapping fields were analysed (magnification ×600).

### Follow-up

The clinical evaluation of patients was performed weekly during the course of RT. Follow-up examinations were performed at 1, 3, 6, and 9 months after completion of RT. Spiral CT-scans of the chest were performed before RT, and at every follow-up examination to monitor morphological changes in lung structure with respect to radiation-induced lung injury and to stage locoregional disease. If distant metastases were suspected, further appropriate staging procedures were performed. Lung function tests (forced expiratory volume; vital capacity; diffusion capacity, etc.) were accomplished before RT, and at every follow-up examination.

### Evaluation of radiation pneumonitis

The occurrence and severity of RP were determined using the LENT-SOMA scale defined by RTOG-EORTC [43]. This evaluation includes three subjective scales (cough, dyspnea, chest pain), two objective scales (radiological abnormalities, lung function) and three management scales (treatment for pain, cough, dyspnea). All single-scale measures range in score from 0 to 4 and the final scoring (lung toxicity: grade 0/I/II/III/IV) is equal to the average of the eight scores. RP is defined as lung toxicity ≥I.

### Evaluation of tumour response

The evaluation of tumour response was performed according to the Response Evaluation Criteria in Solid Tumours (RECIST) guidelines, based on unidimensional measurement of tumour lesions [44]. All baseline evaluations were performed close to the beginning of treatment (≤2 weeks before RT). In all patients, spiral CT-scans of the chest -at baseline and during follow-up examinations- have been used to measure the target lesions selected for response assessment [45]. The response criteria for the target lesions are: complete response (CR): disappearance of all target lesions; partial response (PR): at least 30% decrease; stable disease (SD): neither PR nor PD criteria met; progressive disease (PD): at least 20% increase, or new lesion(s) [44], [45].

### Statistical methods

The patients' clinical characteristics, as well as the disease and treatment-related factors were related to the lung toxicity score using Spearman's correlation coefficient. The prognostic potential of IL-6 and TGF-β1 plasma levels to predict RP was assessed in various forms. Firstly, plasma IL-6 and TGF-β1 levels, respectively, at the end of RT were divided by the corresponding pre-RT values and on a log-scale (base 10) the ratios showed an approximate normal distribution. The ratios (IL-6 end-RT/IL-6 pre-RT; TGF-β1 end-RT/TGF-β1 pre-RT) were related to the lung toxicity score (grade: 0/I/II/III/IV) using Spearman's rank correlation coefficient and the statistical test of zero correlation. The same procedure was repeated for the ratio obtained by replacing the IL-6/TGF-β1 value at the end of RT by the IL-6/TGF-β1 peak value during RT (IL-6 peak-RT/IL-6 pre-RT; TGF-β1 peak-RT/TGF-β1 pre-RT).

The relationship between tumour response after RT and simultaneously measured IL-6 and TGF-β1 plasma levels, respectively, was assessed graphically using a dot plot where each dot refers to one follow-up examination. Statistical significance of differences between the tumour response categories (CR, PR, SD, PD) was obtained by fitting a mixed model using the compound symmetry assumption and the Kenward Roger method. This is an established method to cope with irregular patterns of repeated measurements. Statistical calculations were performed by SAS statistical software. The criterion for statistical significance was p<0.05. All p-values are two-sided.

## Results

### Patient, tumour, and treatment characteristics

52 patients with NSCLC who had tumour specimens available for immunohistochemistry and who appeared for follow-up for at least 6 months after completion of RT/RCT were eligible for this study. The characteristics of the patients are summarized in table 1. The median age was 65.1 years (range 36–85 years). Of the 52 patients, 41 (78.8%) were male and 11 (21.2%) were female. 12 patients (23.1%) had clinical stage I or II and 39 patients (75%) had advanced stage III NSCLC. 28 patients (53.9%) received curative RCT (normo-fractionated radiotherapy with 60–66 Gy and concurrent cisplatin-based chemotherapy); 14 patients (26.9%), not eligible for cisplatin-based chemotherapy, were treated with normo-fractionated RT alone (66 Gy); 10 patients (19.2%) were treated according to a palliative accelerated irradiation regimen (PAIR).

**Table 1:** Patient characteristics.

Characteristic	No. of patients	%
**Age, years**		
Median	65.1	
Range	36–85	
**Gender**		
Male	41	78.8
Female	11	21.2
**Clinical stage**		
I	5	9.6
II	7	13.5
IIIa	13	25.0
IIIb	26	50.0
Unknown	1	1.9
**Histology**		
SCC	27	51.9
AC	21	40.4
Others	4	7.7
**Treatment**		
Curative RCT	28	53.9
Curative RT	14	26.9
Palliative RT	10	19.2
**Radiation Pneumonitis**		
0	31	59.6
I	11	21.2
II	3	5.8
III	6	11.5
IV	1	1.9

### Treatment toxicity

21 patients (40.4%) developed symptomatic RP according to the LENT-SOMA scale. 14 patients showed moderate lung toxicity with grade I in 11 patients (21.2%) and grade II in 3 patients (5.8%). But 7 patients suffered severe lung toxicity with grade III in 6 patients (11.5%) and even grade IV in 1 patient (1.9%) (table 1). In all patients RP was accompanied by worsening of respiratory symptoms with deterioration of lung function parameters. In 19 of the 21 patients with RP, radiological changes were observed in chest CT-scans. 7 patients required corticosteroids and intermittent or continuous oxygen supply. One patient developed a global respiratory failure and needed assisted ventilation; this patient died three weeks later due to pneumonic complications.

For this limited patient population, no significant associations between patients' clinical characteristics (age, gender, smoking history) and the occurrence of RP were observed. Moreover, no disease-related (tumour location, affected lung, clinical stage) or treatment-related factors (concurrent chemotherapy, total radiation dose, radiation fractionation, V20/V30 values) were found to significantly predict the risk of RP in the analysed patient population (Spearman correlation test). However, the total number of patients is certainly to low to perform valid subgroup analysis.

### Cytokine plasma levels at the onset of radiation pneumonitis

**Figure 1:**
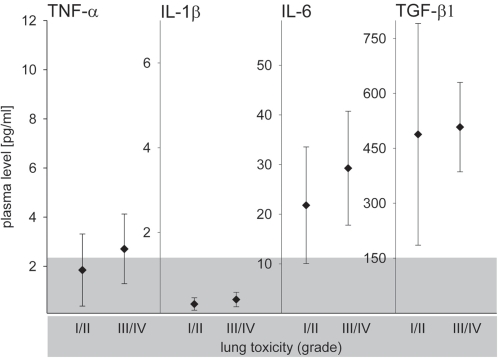
TNF-α, IL-1β, IL-6 and TGF-β1 plasma levels at the onset of radiation pneumonitis (mean values), depicted separately for patients with moderate (grade I/II) (n = 14) and severe lung toxicity (grade III/IV) (n = 7). Error bars represent SEs of the means. Gray-shaded area indicates the normal range of the cytokine plasma levels.

At the onset of RP, plasma levels for IL-6 and TGF-β1 were increased in patients suffering moderate (grade I/II) and severe lung toxicities (grade III/IV) (fig. 1). But no clear correlation between the extent of IL-6 or TGF-β1 elevation and lung toxicity score was observed (fig. 1). In most of the 21 patients suffering lung toxicity, IL-6 and TGF-β1 plasma levels measured at the onset of RP were increased compared to their individual pre-RT values (in 17 out of 21 patients for IL-6 [81.0%]; in 15 out of 21 patients for TGF-β1 [71.4%]) (data not shown). This observation may indicate that the inflammatory process of radiation-induced lung injury is associated with an increased production of IL-6 and TGF-β1 within the irradiation field, which is launched into the blood circulation leading to elevated plasma levels. Plasma concentrations measured for TNF-α and IL-1β, by contrast, exhibited no increased values at the onset of RP (fig. 1).

### Cytokine plasma levels before, during and after completion of RT

**Figure 2:**
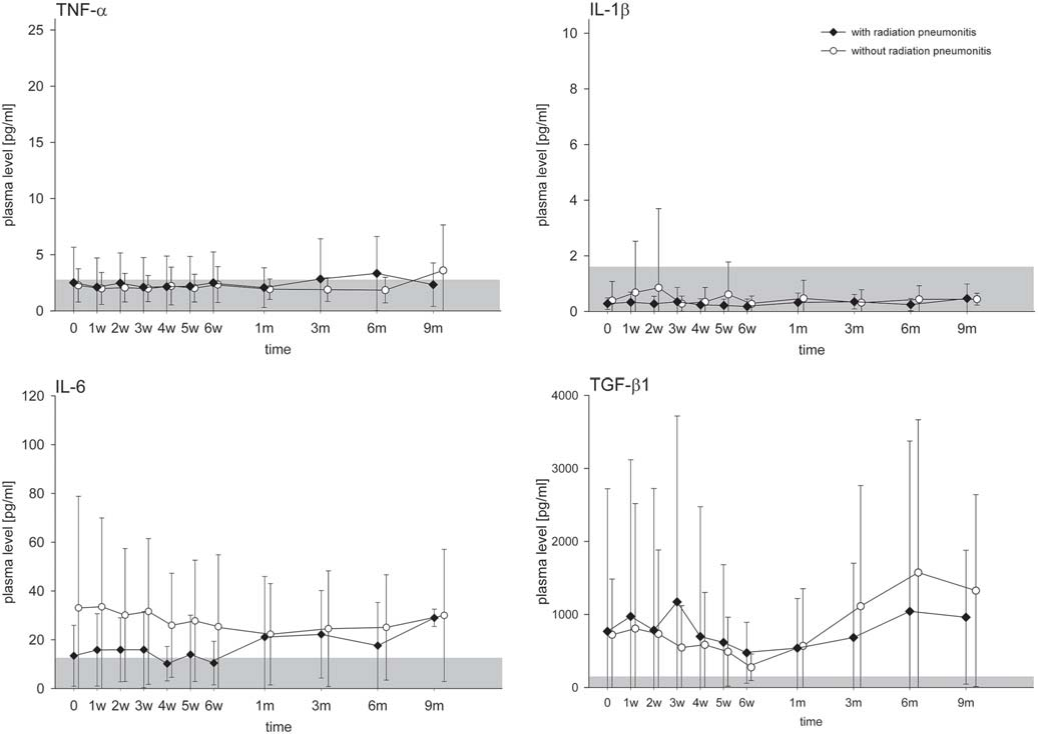
Time courses of TNF-α, IL-1β, IL-6 and TGF-β1 plasma levels (mean values) before (0), during (1–6 weeks) and after radiotherapy (1–9 months), depicted separately for patients *with* (n = 21) and *without* radiation pneumonitis (n = 31). Error bars represent SEs of the means. Gray-shaded area indicates the normal range of the cytokine plasma levels.

In figure 2 the cytokine dynamics are depicted separately for patients *with* (lung toxicity: grade I–IV) and *without RP*. The measured TNF-α and IL-1β plasma levels were within normal ranges (as expected for healthy individuals) at nearly all times and no significant differences between patients with or without RP were observed. For IL-6, in contrast, the plasma levels in patients *not* developing RP were already increased before and during RT. This contrasts to previous studies [20], [21], where higher IL-6 levels were observed in patients suffering RP. After completion of RT during follow-up (1–9 months) both patient groups (*with* and *without* RP) exhibited slightly increased IL-6 plasma levels.

The mean values of the TGF-β1 plasma levels were clearly increased before, during and after RT in both patient groups. A striking observation was the large variation of the TGF-β1 plasma levels in patients irradiated for NSCLC, which ranged from 116 to 9242 pg/ml (fig. 2). The pre-RT TGF-β1 levels were already elevated above the cut-off (⇒150 pg/ml) in 47 of 52 analysed patients (90.4%) (data not shown). For both patient groups (*with* and *without* RP), there was a trend towards a decline of TGF-β1 levels at the end of RT (4–6 weeks) and a clear rise after completion of RT during the follow-up (1–9 months) (fig. 2).

Collectively, no association was observed between the absolute TNF-α, IL-1β, IL-6 and TGF-β1 plasma concentrations measured before or during RT and the risk of RP. Thus, TNF-α, IL-1β, IL-6 and TGF-β1 plasma levels *per se* measured before or during the course of RT did not allow the prospective identification of patients at risk for RP.

### IL-6 and TGF-β1 ratios and the incidence of radiation pneumonitis

**Figure 3:**
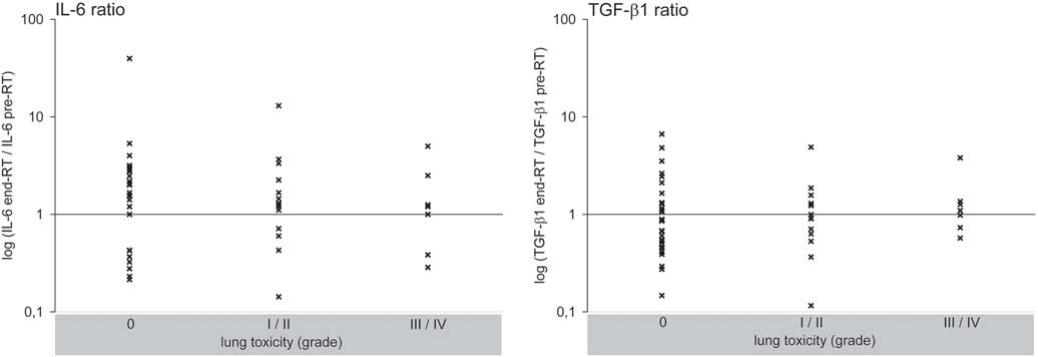
Correlations between the IL-6 and TGF-β1 ratios and the incidence of radiation pneumonitis. For every patient, the ratios of the plasma levels at the end of radiotherapy (end-RT) and before RT (pre-RT) are plotted logarithmically (base 10): relative values >imply an increase (supposed high risk for RP), values <1 imply a decrease of the plasma levels at the end of RT (supposed low risk for RP). The ratios were plotted separately for the patients without radiation pneumonitis (grade 0) (n = 31), and with moderate (grade I/II) (n = 14) and severe (grade III/IV) (n = 7) lung toxicity.

To test the hypothesis that IL-6 and TGF-β1 ratios can be used for the identification of patients at risk as previously proposed in the literature [15], [17], [18], [19], [20], [21], [22], the cytokine plasma levels measured at the end of RT were divided by the corresponding pre-RT values for all patients and correlated with the incidence of RP. In figure 3, these relative values for IL-6 and TGF-β1 are plotted separately on a base 10 logarithmic scale for the patients without RP (grade 0), as well as for patients with moderate (grade I/II) and severe lung toxicity (grade III/IV). Relative values >1 imply an increase (associated with high risk for RP according to the literature), values <1 imply a decrease of the plasma levels at the end of RT compared to the pre-RT levels (associated with low risk for RP) [15], [16], [17], [18], [22]. Figure 3 illustrates that the ratios for IL-6 and TGF-β1 were similarly distributed in all patients irrespective of their suffered lung toxicity. Similar results were obtained for ratios obtained by maximal plasma levels measured during RT divided by the corresponding pre-RT values (data not shown). In summary, we observed no statistically significant correlation between the IL-6 and TGF-β1 ratios and the incidence of RP (end-RT IL-6/pre-RT IL-6: r = 0.08; p = 0.59; end-RT TGF-β1/pre-RT TGF-β1: r = 0.04; p = 0.80).

### Correlation between pre-RT IL-6 and pre-RT TGF-β1 plasma levels and IL-6/TGF-β1 expression in corresponding tumour biopsies

**Figure 4:**
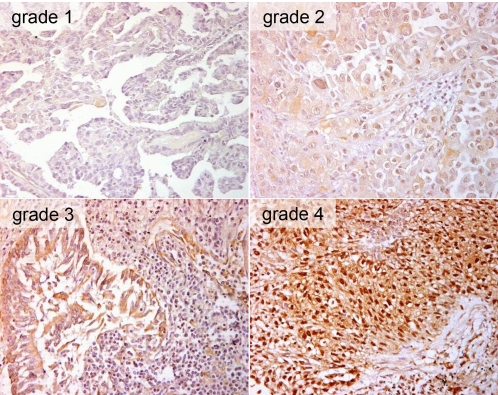
TGF-β1 immunohistochemistry of the tumour biopsies. Quantification of the staining intensity: grade 1 = weak, but detectable focal staining, grade 2 = moderate staining, grade 3 = marked staining, grade 4 = intense staining of the tumour cells (original magnification ×600).

**Figure 5:**
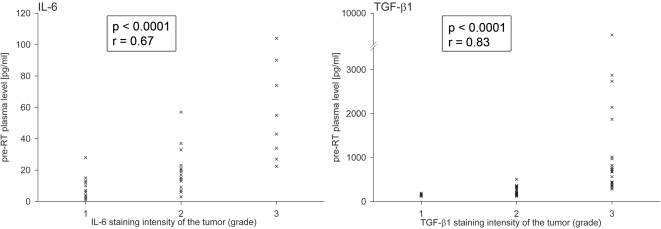
Correlation between the pre-RT IL-6 and TGF-β1 plasma levels, respectively, and the IL-6 and TGF-β1 staining intensity of the corresponding tumour biopsies (grade 1–4) (IL-6: no grade 4 samples). For both cytokines, statistically significant correlations were found between the amount of pre-RT plasma levels and the staining intensity of the corresponding tumour biopsies.

To evaluate the potential impact of tumour-derived cytokine production on circulating plasma levels, the tumour biopsies of the NSCLC patients were immunohistochemically stained for IL-6 and TGF-β1. The 52 analysed tumour specimens revealed a heterogeneous staining pattern for IL-6 and TGF-β1 with variably intense cytoplasmatic and/or nuclear staining of the tumour cells. Examples of the TGF-β1 immunohistochemistry of tumour biopsies are presented in figure 4. Compared to the rather intense staining for TGF-β1 in most of the analyzed tumour biopsies, the IL-6 staining of the tumour specimens was generally weaker. In figure 5 the pre-RT plasma levels for IL-6 and TGF-β1, respectively, were plotted against the staining intensity of the corresponding tumour specimens for every patient. For both cytokines a statistically significant correlation was found between the amount of pre-RT plasma levels and the staining intensity of the corresponding tumour biopsies, suggesting that -irrespective of the subsequent irradiation- the IL-6 and TGF-β1 plasma levels in NSCLC patients were influenced to a great extent by the cytokine release of their tumours (IL-6: r = 0.67, p<0.0001; TGF-β1: r = 0.83, p<0.0001).

### Correlation between tumour response and IL-6/TGF-β1 plasma levels assessed at the same time-points during follow-up

**Figure 6:**
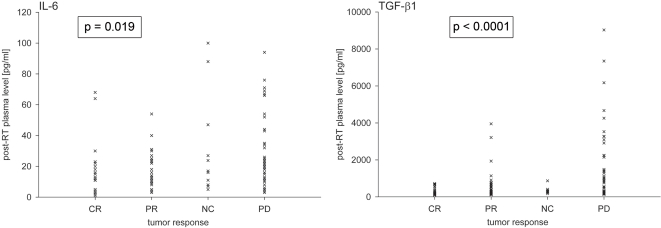
Correlation between the tumour response (CR = complete remission; PR = partial remission; NC = no change; PD = progressive disease) and the IL-6 and TGF-β1 plasma levels assessed at the same time-points during follow-up. For both cytokines, statistically significant correlations were observed between the tumour response categories and IL-6 and TGF-β1 plasma levels.

To find out whether increased IL-6 and TGF-β1 plasma levels after completion of RT originate from the tumour, IL-6 and TGF-β1 plasma concentrations measured at defined time-points during follow-up were plotted against the concurrently determined tumour responses of every patient (fig. 6). The evaluation of the tumour response (CR, PR, SD, PD) was performed by measurement of tumour lesions in CT-scans of the chest acquired during follow-up (1, 3, 6, and 9 months). As shown in figure 6, statistically significant correlations were observed between the tumour response and IL-6 and TGF-β1 plasma levels, respectively, assessed at the same time-points during follow-up (p = 0.019 and p<0.0001, trend tests based on mixed model analysis). Noteworthy, for every patient at least three evaluation time-points during follow-up (1, 3 and 6 months after RT) were included in figure 6.

## Discussion

In the last years many efforts focused on the determination of clinically useful indicators (physical and biological parameters) to discriminate between patients with high and low risk of pulmonary complications after thoracic irradiation for NSCLC. The ability to select patients based on their relative risk for RP would allow the safe delivery of higher irradiation doses in suitable patients, with possibly improved local control and survival. Recent insights into the pathogenesis of radiation-induced lung injury revealed a number of biological factors which are not only involved in the development and course of the disease but may also serve as a predictive marker for RP. A possible link between rising cytokine plasma levels during RT and the subsequent development of RP has been discussed in the literature [15], [16], [17], [18], [19], [20], [21], [22]. However, two potential sources of cytokine plasma levels have been suggested: first, the gradually accumulating normal tissue damage leads to an increased cytokine production within the irradiation field, which is launched into the blood circulation. Second, the tumour may produce variable amounts of cytokines and, during the course of RT, cytokine plasma levels may decline, stay elevated or even increase depending on the tumour response to treatment [7], [46].

In the present study, we investigated prospectively the dynamics of pro-inflammatory (TNF-α, IL-1β, IL-6) and pro-fibrogenic (TGF-β1) cytokine levels in patients irradiated for NSCLC to ascertain their prognostic value to predict RP. Furthermore, an attempt has been made to evaluate the potential impact of a tumour-derived cytokine production on circulating plasma levels which may impair the prospective identification of patients at risk for RP. The results of our study did not confirm that TNF-α, IL-1β, IL-6 or TGF-β1 plasma levels, neither their absolute nor any relative values, may identify patients at risk for RP. Our results are in accordance with the studies of Novakova-Jiresova *et al.* and De Jaeger *et al.*, demonstrating that increased TGF-β1 plasma levels at the end of RT are not independent risk factors for developing symptomatic RP [47], [48]. Moreover, we could show that IL-6 and TGF-β1 is produced in the tumour tissues of most NSCLC patients to variable degree. The clear correlation between the pre-RT plasma levels of IL-6 and TGF-β1 and the respective cytokine production in the corresponding tumour biopsies suggests that the elevated plasma levels in NSCLC patients result from the over-production in their tumours. Furthermore, our data indicate a significant association between the IL-6 and TGF-β1 plasma levels and the individual tumour responses assessed at the same time-points during follow-up. Based on our findings we conclude that the tumour is a major source of circulating cytokines in patients receiving radiotherapy for advanced NSCLC and this tumour-derived cytokine production may impair the prospective identification of patients at risk for RP.

Numerous studies emphasize the important role of IL-6 and TGF-β1 in the pathophysiology of cancer [23], [33], [49]. In NSCLC cell lines IL-6 has been shown to be involved in tumour cell proliferation, in tumour promotion and progression [50], [51], [52], [53], [54], [55], [56]. In NSCLC patients elevated IL-6 plasma levels were related to tumour size, increased with tumour progression and have been associated with a worse prognosis [57], [58], [59]. In late stages of tumour progression, TGF-β1 is known to stimulate invasion, angiogenesis and metastasis and to inhibit the immunosurveillance [60], [61], [62], [63], [64]. Increased plasma concentrations of TGF-β1, due to an over-production in the tumour tissue, have been reported in patients with NSCLC and various other cancers [36], [65], [66]. Several clinical reports have highlighted the prognostic importance of TGF-β1 in a variety of tumours, including NSCLC, suggesting that circulating plasma levels are correlated with the extent of disease and disease recurrence [35], [66], [67], [68], [69].

In summary, our results indicate that monitoring plasma concentrations of TNF-α, IL-1β, IL-6 and TGF-β1 does not allow a predictive risk assessment for RP in patients irradiated for advanced NSCLC. In contrast, IL-6 and TGF-β1 plasma levels measured in patients with advanced NSCLC depend to a great extent on the cytokine production in their tumours. Additional release of IL-6 or TGF-β1 into the blood circulation as a result of radiation-induced lung injury is often superimposed by the variable cytokine production of the tumour and thus will be difficult to detect. Hopefully, future studies will identify reliable predictors of adverse radiotherapy effects, and thus provide the opportunity to individualize the treatment to improve the therapeutic outcome.
